# Reconstruction of Barely Visible Impact Damage in Composite Structures Based on Non-Destructive Evaluation Results

**DOI:** 10.3390/s19214629

**Published:** 2019-10-24

**Authors:** Angelika Wronkowicz-Katunin, Andrzej Katunin, Krzysztof Dragan

**Affiliations:** 1 Department of Fundamentals of Machinery Design, Silesian University of Technology, Konarskiego 18A, 44-100 Gliwice, Poland; angelika.wronkowicz-katunin@polsl.pl; 2 Air Force Institute of Technology, Airworthiness Division, Ks. Bolesława 6, 01-494 Warsaw, Poland; krzysztof.dragan@itwl.pl

**Keywords:** barely visible impact damage, aircraft composites, non-destructive testing, ultrasonic testing, X-ray computed tomography, image processing, damage reconstruction

## Abstract

The occurrence of barely visible impact damage (BVID) in aircraft composite components and structures being in operation is a serious problem, which threatens structural safety of an aircraft, and should be timely detected and, if necessary, repaired according to the obligatory regulations of currently applied maintenance methodologies. Due to difficulties with a proper detection of such a type of damage even with non-destructive testing (NDT) methods as well as manual evaluation of the testing results, supporting algorithms for post-processing of these results seem to be of a high interest for aircraft maintenance community. In the following study, the authors proposed new approaches for BVID reconstruction based on results of ultrasonic and X-ray computed tomographic testing using authored advanced image processing algorithms. The studies were performed on real composite structures taking into consideration failure mechanisms occurring during impact damaging. The developed algorithms allow extracting relevant diagnostic information both from ultrasonic B-and C-Scans as well as from tomographic 3D arrays used for the validation of ultrasonic reconstructed damage locations, which allows for a significant improvement of the detectability of BVID in tested structures. The developed approach can be especially useful for NDT operators evaluating the results of structural NDT inspections.

## 1. Introduction

Polymeric composite materials, due to their numerous advantages, have found a very wide application in many branches of industry, especially the automotive, aircraft, aerospace, or marine. They have become competitive alternatives to traditional metallic materials mainly due to significant weight savings, greater strength and stiffness, and corrosion resistance.

However, due to their complex nature, anisotropy, non-homogeneity, and construction with interfaces, polymeric composites are characterized by low performance at high temperature, poor through-the-thickness properties, and low resistance to impact loadings [1]. Composites are subjected to many factors that may cause damage throughout the service life of a composite component (e.g., impacts, static overloads, fatigue, overheating, or hydrothermal effects [2]). To the in-service damage types one can include delamination, cracks, ingress of moisture, buckling or fracture of fibers, failure of bonds or the matrix/fibers interface, and impact damage. In many industrial reports and scientific publications, it is reported that a particularly common and complex problem is low energy impact damage, which is the source of the most concern, especially in the case of the aircraft elements (where they are caused by, e.g., runway debris, hailstorms, or dropped maintenance tools). Low energy impacts are invisible or barely visible on the impacted surface, whereas they may cause remarkable internal damage in a form of tree root of matrix cracks and propagating delamination [3], which lowers the composites’ residual strength. Therefore, such impact damage is classified as so-called barely visible impact damage (BVID). BVID represents a significant threat to the composite structure integrity and may result in failure before the end of its expected life period [4]. As reported in the survey of structural health monitoring technology and usage by FAA Airworthiness Assurance Center and Sandia National Laboratories [5], impact damage identification is one of the most important current needs of the aerospace industry regarding Structural Health Monitoring (SHM) systems design and implementation.

Commonly applied damage tolerance methodology involves allowing composite components with internal damage to be operated if certain conditions are met [6]. If the damage in the component does not weaken the structural integrity, the structure is considered damage tolerant. A maintenance program must be implemented in order to identify the damage and monitor its progression, which allows for an adequate reaction before an unacceptable reduction of the residual life of this structure. For this purpose, periodic inspections with the use of non-destructive testing (NDT) methods are performed, which allows for the identification of damage extent and the observation of its growth. This procedure is very important due to a very complex nature of initiating and propagating of composite damage [7].

One of the most commonly applied NDT methods during routine inspections of aircraft composite structures is ultrasonic testing (UT). In comparison to other commonly used NDT methods, UT is the leader concerning the capabilities for the detection, sizing, and depth estimation of damage [8]. The reason for favoring ultrasonic inspection is its high sensitivity to various damage types commonly found in composites [2] as well as the FAA and other authorities’ approval for this NDT method. Using this method, due to its volumetric nature, it is possible to determine a geometry of internal damage and its surface area. However, depending on specific UT method applied during an inspection, various complications and obstacles may appear, which can be generally referred to technical-related factors. These factors include undesired ultrasonic wave interactions, like reflections, refractions, diffractions, scattering, and attenuation; existence of so-called dead zones, which make it difficult to detect near-surface damage; and finally a relation between the scanning beam diameter and a size of a discontinuity to be detected, i.e., the beam diameter defines a limitation of the detectability. A wide discussion of these factors can be found in [9]. Obviously, all of these technical factors influence negatively on the quality of NDT results obtained from inspections, and, in consequence, on the quality of the evaluation of structural condition and its residual life.

Besides the above-presented technical factors, various human-related factors are of high importance, since they often determine the quality of the evaluation and prognosis of structural properties. One of the crucial factors influencing the results is that raw UT results are often processed manually, which is a typical practice in aircraft inspections mainly due to complexity of the obtained data, and thus, the impossibility of a substitution of an inspector by computer algorithms. During industrial ultrasonic inspections, e.g., during testing of aircraft structures, the UT results have to be analyzed and interpreted by a certified expert, having appropriate knowledge about the specifics of UT, mechanics of composite structures, and the form of the tested element (including locations of bolt connections, embedded elements, etc.). This often leads to oversights and misinterpretations due to the physical impossibility of the detection of structural changes based on UT raw results in some cases. Therefore, in order to increase the efficiency of the UT results analysis, there are some methods described in research studies, which can be found in the literature, that are focused on applying image processing techniques to the ultrasonic scans in order to extract more diagnostic information than is possible using commercial software dedicated to UT.

A 3D damage reconstruction based on ultrasonic scans is one of the presently undertaken goals in the area of UT results visualization, which can provide a very significant support to the inspection operator. The necessity of 3D reconstruction of UT results to enhance damage visualization was noticed and communicated more than 30 years ago, when UT systems started to be digitalized due to the development of computer technologies [10], however, there is still so far little work devoted to the development of a universal method of damage extraction and 3D reconstruction based on ultrasonic scans. One possible reason is that the real composite structures, e.g., a vertical stabilizer of aircraft, affected by damage, reflects an ultrasonic response with a very high degree of complexity, which is difficult to be interpret properly. The early approaches used for the evaluation of UT data and its 3D visualization were based on primary image segmentation methods [11] and evaluation of statistical features [12]. The approach of 3D reconstruction of UT data found an application in various branches, e.g., Yeh and Liu [13] used 3D rendering techniques as a processing tool for 3D reconstruction of cracks in concrete structures. The 3D rendering of B-Scans of carbon fiber-reinforced polymeric (CFRP) composite structures with impact damage were presented in [14] using the thresholding rules, which allowed for significant increasing of the evaluability of this type of damage inside a structure. One of other approaches of 3D reconstruction of composite defects based on UT is presented in [15], which was based on image filtering and thresholding. Later, the same research group presented the method of UT image segmentation in order to automatize damage evaluation process of CFRP composite structures [16]. Several attempts to processing of raw results of UT and their further 3D reconstruction was presented by Smith in his dissertation [17]. Grandin and Gray performed 3D reconstruction of ultrasonic C-Scans via segmentation using two algorithms, thresholding and binomial hypothesis tests, which allowed extracting desired diagnostic information [18]. Attempts to 3D reconstruction of ultrasonic C-Scans were undertaken by Dragan and his research team. For example, in [19] the authors used an edge detection algorithm for the extraction and further reconstruction of impact damage from C-Scans, while in [20,21] they used a set of advanced filtering using Difference of Gaussians and Wiener filters, adaptive thresholding, and morphological operations in order to obtain high-quality 3D reconstruction of UT data. Another approach was proposed by Segreto et al. [22], where the authors applied signal processing procedures to particular UT waveforms representing particular measurement points, and then the processed waveforms were composed back and visualized in 3D. A variety of processing algorithms for improving defect detectability and enhancing visualization of defects based on ultrasonic B-Scans were presented in [23]. The authors used cross-correlation, wavelet, and Hilbert–Huang transforms for enhancing identification of a composite structure cut out from the wind turbine blade with artificially introduced defects. The latest published results in 3D reconstruction of C-Scans were addressed to medical applications [24]. However, the applied processing algorithms, which are based on K-nearest neighbor filtering in this case, are the same in principle.

Previous studies of the authors of the following paper include numerous processing and reconstruction algorithms developed for the enhancement of damage detectability and improvement of decision-making processes. The first studies on the enhancement of damage detectability by its 3D reconstruction were introduced in [25], where the authors proposed the approach based on multilevel Otsu thresholding and morphological processing of ultrasonic C-Scans of a vertical stabilizer of an aircraft. A 3D pseudo-reconstruction algorithm based on thresholding of values obtained from C-Scans was demonstrated in [26]. A preliminary 3D reconstruction algorithm of C-Scans with filling gaps resulting from hiding the damage extent at subsequent layers by flaws located above this damage was presented in [27]. However, the nature of impact damage is not always well represented by such a reconstruction by filling the hidden damage areas, therefore, it was decided to develop similar procedures of 3D reconstruction and visualization of such a type of damage in composite structures using B-Scans as the input data. Appropriate comparative studies for the obtained results from C-Scans and B-Scans, as well as a validation of these results using 3D reconstructed X-ray computed tomography (CT) data, were presented in [28]. Although, the effectiveness of the proposed algorithms was much higher than in the previous studies, i.e., one can distinguish damage in particular layers of a tested composite structure, these algorithms were still manual or semi-automatic, and, due to the lack of their universality, they cannot be applied without advanced tuning for a specific case.

The aim of this study is the development of universal and automatic algorithms for processing and reconstruction of ultrasonic data obtained using B-and C-Scan testing techniques. In order to verify the quality of the obtained reconstructed data from UT, the validation was performed based on comparison with X-ray CT reconstructions. Since X-ray CT is the most accurate NDT method, which additionally provides raw 3D data for a tested structure, it can be assumed as the reference data for such a comparison. In this study, all the processing and reconstruction algorithms for both UT and X-ray CT data are based on newly developed authored methods with an improved impact damage detectability accuracy and automatization. 

One of the purposes of the reconstruction of ultrasonic scans is the simplification of the evaluation process of structural damage performed by NDT operators. However, the accurately reconstructed damage sites give much more possibilities of their application. According to the currently developed trends in structural condition evaluation and maintenance, e.g., the condition-based maintenance (CBM) program, besides the structural diagnostics, which includes three main steps (damage detection, localization, and identification), it provides also the fourth step—prognostics. It should be highlighted that prognostics deals with fault prediction before it occurs, i.e., estimation of the residual strength of a tested element, which is not possible when applying the diagnostic methods only. The reconstructed damage from UT inspections, including the exact shape of damage, can be used for structural integrity evaluation and prognosing the condition of composite structures via inverse finite element (FE) models, which is the main goal of the currently implemented research project. The presented results are a part of this ongoing research.

## 2. Failure Mechanisms of BVID in Composite Structures

Despite the excellent mechanical properties of composite materials and structures made of them, they reveal high vulnerability to impacts, especially CFRP structures, which can be classified due to the impact velocity to low-, medium-, and high-velocity impacts. Although medium- and high-velocity impacts cause visible impact damage (VID), which is well detectable by observing a damaged region with a naked eye (surface cracks, penetration, etc.), the low-velocity impacts (LVI) result in structural damage, which can be detected using special NDT equipment, since the signs of this damage are invisible or barely visible. This type of damage is called barely visible impact damage (BVID). Due to the lack of clear indications of damage, its presence is very dangerous for structures being in operation and subjected to any kind of loading, since their presence can be overseen. Therefore, as it was mentioned in the previous section, currently implemented maintenance methodologies of composite structures, especially in the aircraft industry, require periodic inspections of such structures in order to detect such a type of damage timely and monitor it appropriately.

In aircraft structures, BVID may occur at various operation circumstances, including hailstorms and bird strikes during in-flight conditions, stone lofting during takeoff or landing, and tool-dropping during ground maintenance [29,30]. The failure mechanisms of impact damage are usually very complex, and can be classified into four main categories: delamination, matrix cracking, fiber breakage, and matrix indentation (see, e.g., [1]), where only delamination and matrix cracking are usually attributed to BVID due to the LVI [31].

The failure mechanism of composite structures subjected to BVID is highly complex due to numerous interactions between components of a composite as well as the appearance of different types of stresses and their redistribution. Such a complex mechanism is a result of energy dissipation during LVI, in contrast to high-velocity impacts, where the impact energy is concentrated on a small area. During these dissipation processes, most of the dissipated energy causes deformations within the elastic range, while a part of this energy results in fracture of the resin and reinforcement.

**Figure 1:**
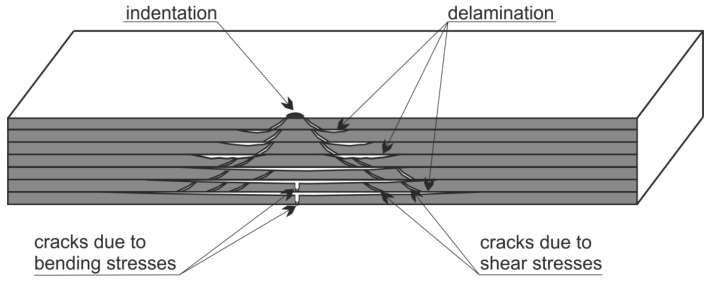
A scheme of barely visible impact damage (BVID) in a composite structure.

As it was mentioned before, the indications on the surface of a composite structure after LVI might be barely visible or even invisible, while inside this structure, extended damage usually appears. Interestingly, the geometric structure of BVID has a form of a truncated cone, and is called the pine tree distribution damage [32,33] (see Figure 1). This characteristic shape is a result of the simultaneous action of tensile, compressive, and shear stresses during LVI.

The true distribution of various types of damage occurring during LVI is difficult to predict due to numerous factors influencing on the failure process, including a shape of impactor, materials composition, its type and properties (i.e., the susceptibility to delamination under LVIs depends, for example, on a material of reinforcing fibers), boundary conditions, a thickness of an impacted structure, and many others. However, one can distinguish several main fracture mechanisms which appear in the majority of such cases. The indentation or crushing (depending on the type of a composite) is a type of damage with negligible influence on the structural stiffness and integrity, and usually it is the only visible sign on an impacted surface. The matrix cracks appearing in a composite structure due to LVI are generally of two types: skew cracks occurring at shear stresses and transverse cracks occurring under the influence of bending stresses (see Figure 1). The appearance of skew cracks is a result of the action of high transverse shear stresses/compressive bending strains and its inclination is about 45° with respect to the impacted surface [34]. The second type of the occurring cracks results from reaching critical in-plane normal stresses/tensile bending strains related to flexural deformations of an impacted structure [35], which achieve the highest values on the opposite side of a structure with respect to the impacted surface. This is the reason of an accumulation of such cracks in bottom layers of a composite. The last type of damage is delamination occurring under the influence of shear stresses, which are triggered by matrix cracks of both types due to the stress concentration at their crack tips, when reaching the matrix/reinforcement interface. Delamination, due to a low resistance of composites for crack propagation in a translaminar direction, may reach large areas compared to the initial indentation. Therefore, delamination is the most dangerous type of structural damage occurring at LVI. In contrast to cracks, delamination may significantly reduce the compressive strength of a composite structure, which may have serious consequences for the load bearing capacity of an aircraft composite structure.

In practice of aircraft ground maintenance, regardless a low influence of BVID on the overall structural stiffness and integrity in general, this type of damage remains one of the most dangerous, since it cannot be detected without a specialist equipment used for advanced NDT. This creates a possible situation where BVID can be left undetected, and may grow under operational loading, which, in consequence may lead to failure of the entire structure. Therefore, the development of appropriate methods of the estimation of BVID extent and the investigation on its influence on structural performance and behavior under operation loading is of high importance for the safety of these structures.

## 3. Experimental Setup of NDT Studies

The evaluation of BVID and further development of algorithms for damage reconstruction was performed on a CFRP structure subjected to controlled impact loading. In order to obtain valid results, the reconstruction was performed based on data acquired from three approaches to NDT applied to the tested specimen.

### 3.1. Specimens and Introduction of Impact Damage

The CFRP specimen used in this study was purchased from the Dexcraft s.c. (Helenów, Poland). It was manufactured in the VARTM resin infusion technology using 2 × 2 twill weave carbon fabric as the reinforcement and epoxy resin. The nominal dimensions of the specimen were as follows: 100 × 100 × 2.5 mm (width × length × thickness).

**Figure 2:**
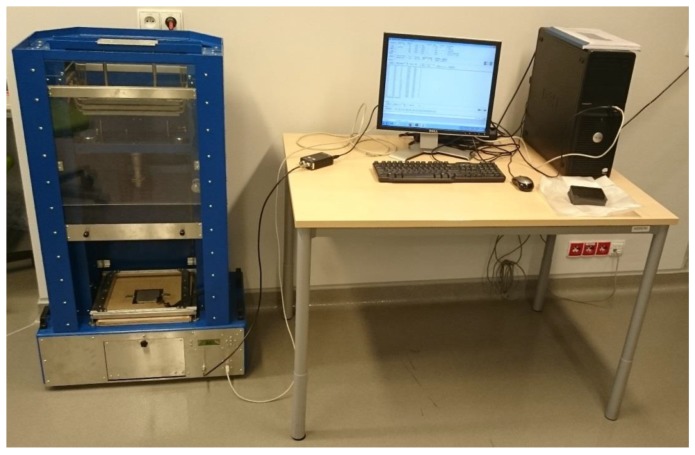
Experimental setup for the introduction of BVID.

**Figure 3:**
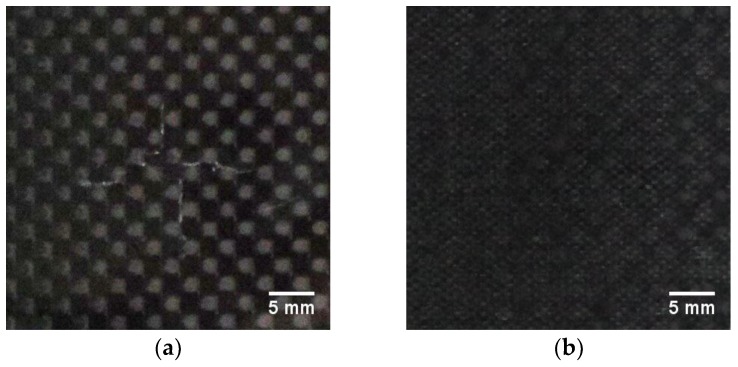
The carbon fiber-reinforced polymeric (CFRP) specimen with impact damage: (**a**) a top view; (**b**) a rear view.

The impact damage was introduced using the in-house drop weight testing machine (see Figure 2), which specification can be found in [36]. A hemispherical impactor with a diameter of 10 mm was used for introducing impact damage with the energy of 20 J. The structure after the impact is presented in Figure 3.

A visual observation of the impacted specimen (see Figure 3) allowed for the detection of barely visible indication of damage only on the top surface of the specimen in a form of a cross-shaped surface crack and a very shallow indentation.

### 3.2. Ultrasonic Testing

The UT was performed in the NDT lab of Air Force Institute of Technology in Warsaw (Poland). The UT parameters were selected based on the previous studies of the authors presented in [9]. The tests were performed in two ultrasonic modes: B-Scans and C-Scans.

**Figure 4:**
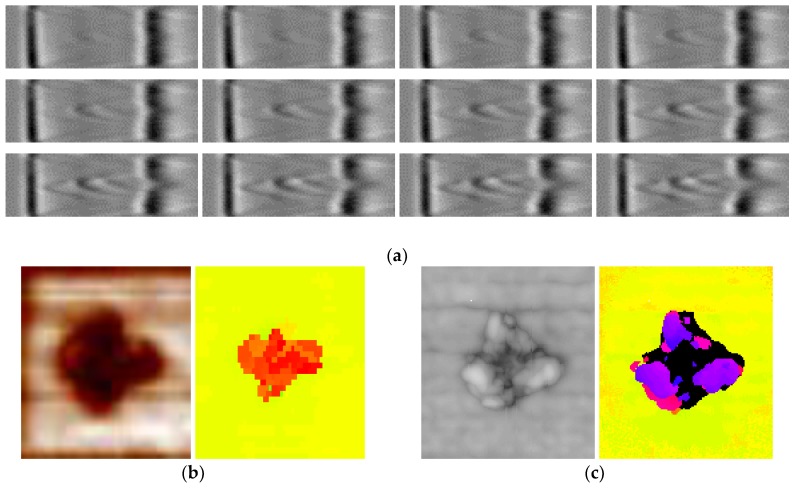
Ultrasonic testing (UT) results: (**a**) the representative set of UT B-Scans (nos. 54÷65 from 170 in total); amplitude and Time-of-Flight (ToF) C-Scans obtained using (**b**) phased-array, and (**c**) pulse-echo methods.

For obtaining B-Scans the phased-array UT method was used. In this case, the tests were performed using the FlawInspecta^®^ scanner with a 128-element phased array transducer and a frequency of 5 MHz. The obtained scanning resolution was of 0.508 mm per pixel. A representative set of the acquired B-Scans is presented in Figure 4a. Since C-Scan can be extracted based on the raw data collected, the resulting C-Scan for this test was also obtained (both in the amplitude and Time-of-Flight (ToF) modes) and is presented in Figure 4b.

The C-Scan used for the reconstruction was obtained using the pulse-echo UT method with the use of the MAUS^®^ water-coupling system. The test was performed using a single straight-beam probe with a delay line with a frequency of 5 MHz, and scanning resolution of 0.254 mm per pixel. The obtained ultrasonic C-Scan, also in the amplitude and ToF modes, is presented in Figure 4c.

The results obtained using both UT techniques indicate the presence of multiple delamination between the composite layers at several specimen depths. As mentioned before, a disadvantage of the presentation of the results in the C-Scan mode is that damage areas located near the top surface of the tested object cover damage fragments being below them. Thus, this is typical for impact damage that there is a partial loss of the diagnostic information visible in the C-Scans. Moreover, the black areas visible in the ToF C-Scan in Figure 4c represent the lack of signal values captured in these positions, which, in this case, correspond to damage located in the ultrasonic transducer dead zone during UT, i.e., very close to the top surface of the specimen. The application of the delay line in the pulse-echo method allowed obtaining additional information about BVID which was partially masked in the results obtained using the phased-array method (due to the interface noises). The results of damage reconstruction proposed in this paper will allow for performing a thorough comparative analysis of the character of the resulting impact damage and observing more dependences between the analyzed types of NDT results.

### 3.3. X-ray Computed Tomography

**Figure 5:**
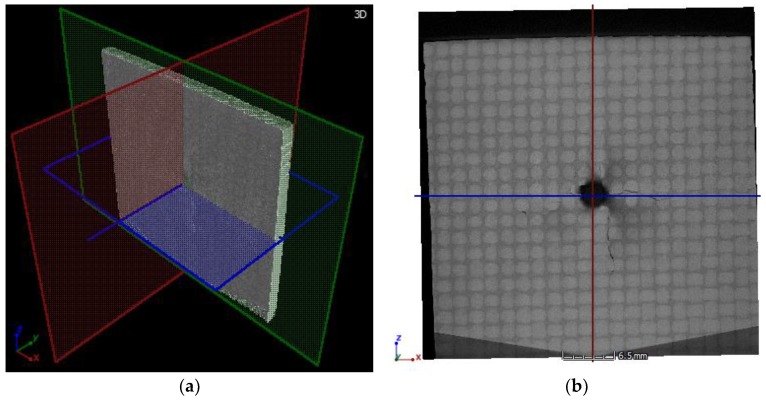
The X-ray computed tomography (CT) results: (**a**) a 3D view; (**b**) an exemplary front section.

The X-ray CT tests were performed by the Machinefish Materials & Technologies Sp. z o.o. Sp. k. (Wrocław, Poland) using the Nikon/Metris XT H 225 ST Computed Tomography System. In order to obtain possibly the highest resolution of the X-ray CT results, the specimen was cut to a smaller section containing the impact damage with surrounding areas, which resulted in reducing the specimen’s dimensions to 40 × 70 × 2.5 mm with the scanning area of 40 × 40 × 2.5 mm, and obtaining the image resolution of ca. 0.022 × 0.022 mm^2^ per pixel. The images (X-ray CT slices) used in this study were generated every 0.01 mm in front section (starting from the top view of the specimen, see Figure 5).

## 4. Development of Algorithms for Impact Damage Reconstruction and Representative Results

For all the obtained types of NDT results, the new processing methods were developed in order to automatically detect the damaged areas and visualize them in 3D properly with respect to the real specimen’s dimensions. These algorithms were implemented using Matlab^®^ and are described below together with the results obtained for the considered specimen case.

### 4.1. Reconstruction Algorithms for UT B-Scans

**Figure 6:**
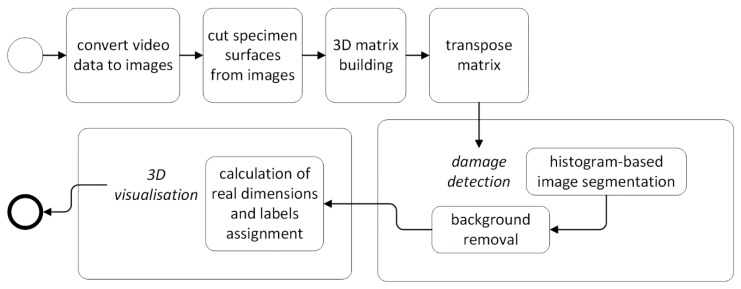
A general reconstruction algorithm for UT B-Scans.

A general scheme of the reconstruction algorithm developed for processing of UT B-Scans is presented in Figure 6. The details on the particular steps of the algorithm are described below.

Firstly, the B-Scans, which were acquired in a form of video data, are converted to images that correspond to the ultrasound reflections in adjacent transverse cross sections of the specimen (see Figure 4a). The darkest areas (i.e., of the highest amplitude values) observable in Figure 4a reflect the specimen’s top and bottom surfaces (seen as the vertical lines) and areas with delamination (the reflections distinguishable between the surfaces).

**Figure 7:**
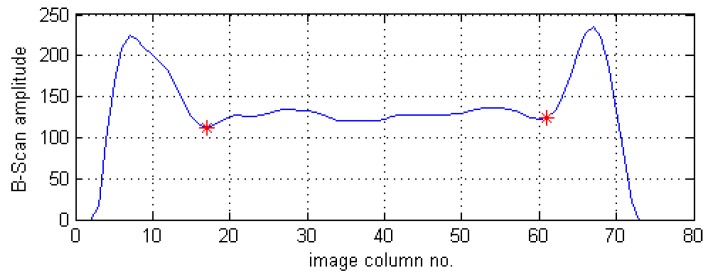
Amplitude values of an exemplary row of a B-Scan and detected minima.

The next step relies on an automatic removal of the B-Scans’ sections that represent the signal reflections from the specimen’s boundary surfaces. For this purpose, for each row of processed B-Scans the extreme positions of surfaces are found by finding two minima values that are nearest (towards the internal direction) from two found biggest maxima values (see an example in Figure 7). The average integer values calculated from all pairs of the minima values found for all the B-Scans’ rows are used to cut all the B-Scans (these minima correspond to the numbers of image columns). 

Afterwards, the B-Scan images after cutting are set together in sequence to create a 3D matrix, which is then transposed from [X Y Z] to [X Z Y] data direction to form data which are more easily manipulated. Namely, this step allowed for changing the representation of particular images from the transverse to the longitudinal cross sections of the specimen, which enables the observation of particular delaminated areas in a planar view.

**Figure 8:**
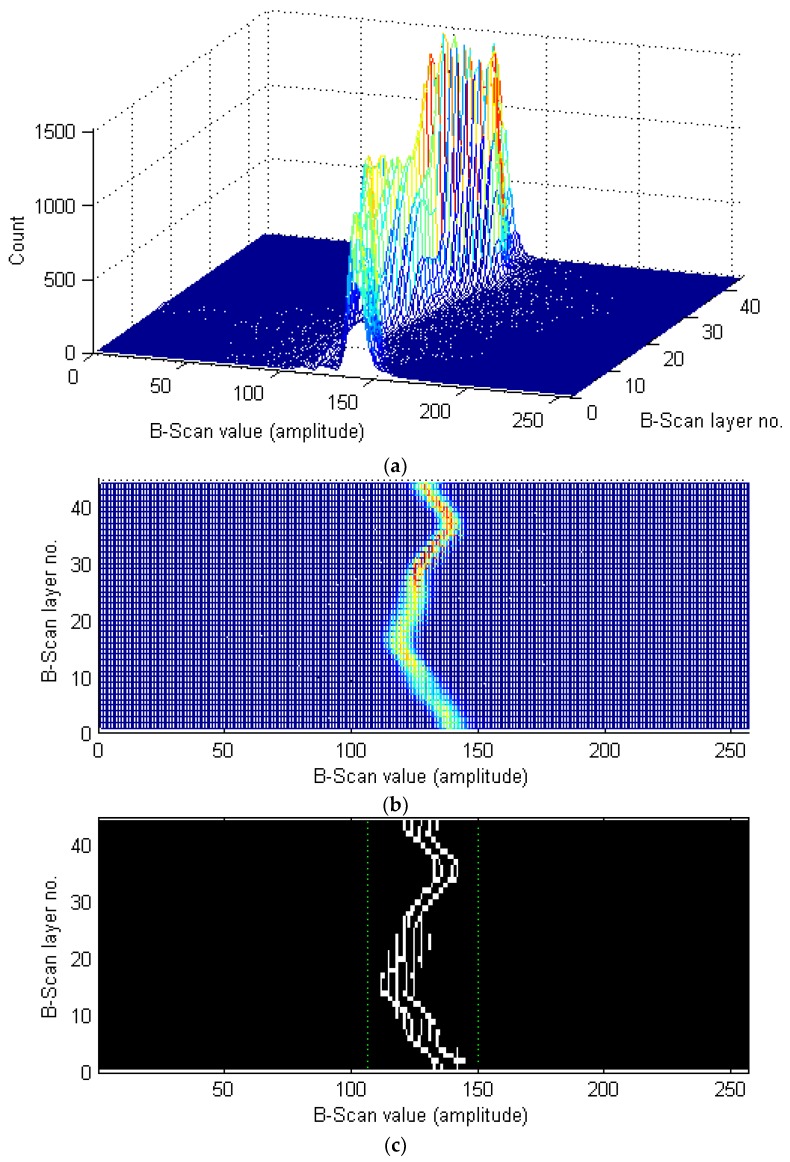
A set of histograms of a series of B-Scan layers: (**a**) a 3D view; (**b**) a top view; (**c**) detected edges and thresholds (shifted).

The next step, connected with damage detection, is based on a new histogram-based image segmentation method. This method relies on automatically finding values of two thresholds used to segment an image, which allows for the separation of the image regions representing damage from the background (i.e., healthy regions of the specimen). This method consists in the extraction of image histograms for all the B-Scan layers (images) and setting them together in sequence (see Figure 8a), which illustrates the amplitude values with the highest counts as the ones representing the background regions, whereas the least frequently occurring amplitude values are considered to be anomaly, i.e., damage. In order to detect the thresholds, the set of histograms is processed as an image (see Figure 8b) to automatically find edges using the Prewitt edge detection method (see the binary result in Figure 8c). This step allows for easy finding the two extreme positions (corresponding to the amplitude values) of the detected edges and using them directly as the thresholds for the image binarization, or in the case of noisy data, optionally, thresholds with a correction shift by a selected number of pixels (as marked in Figure 8c, thresholds shifted from the edges by five amplitude units).

**Figure 9:**
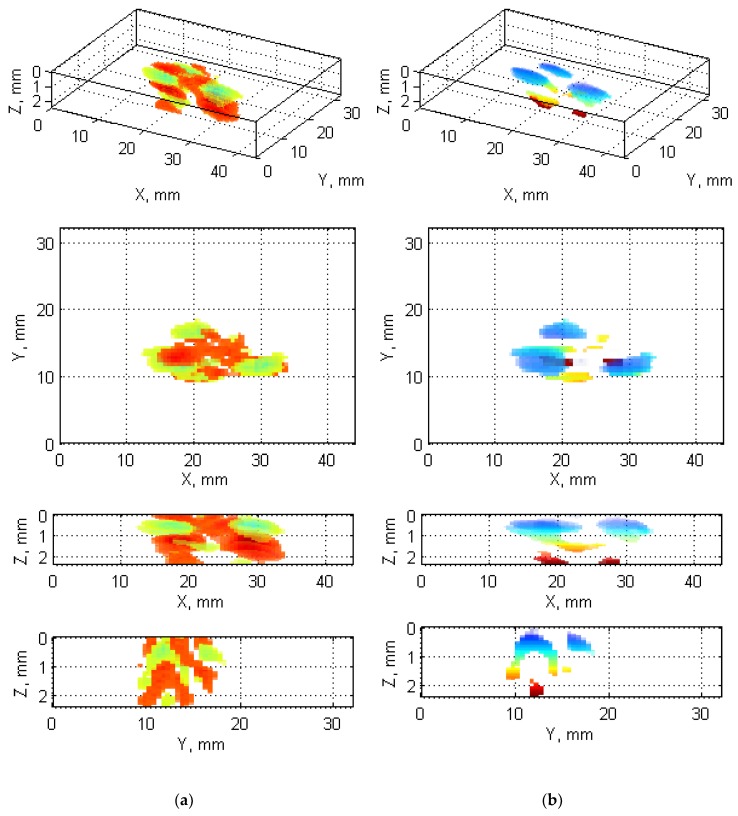
Visualization of the reconstructed damage based on UT B-Scans: (**a**) all regions beyond two thresholds; (**b**) regions below the first threshold.

Subsequently, the background (i.e., regions of the amplitude values between the two thresholds) is removed and damage is visualized (see Figure 9a) after assigning the axes values and labels corresponding to the real specimen dimensions. This assignment is possible owing to calculations of the pixel-millimeters ratios and input data with values of the scanning resolution and the thickness of the tested element. However, it was noticed that the regions of higher amplitude values (above the second threshold) are the reflections from the composite layers around the damage regions and thus, leaving only the regions below the first binarization threshold (see the result in Figure 9b) may return more clear visualization of the damage.

### 4.2. Reconstruction Algorithms for UT C-Scans

**Figure 10:**
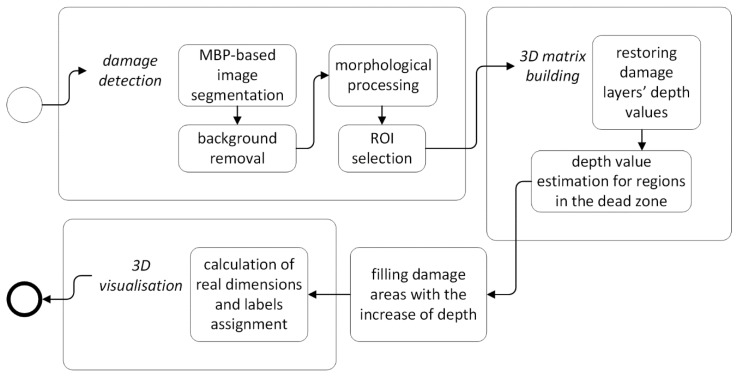
A general reconstruction algorithm for UT C-Scans.

A general scheme of the reconstruction algorithm based on UT C-Scans is presented in Figure 10. This procedure is applied to ToF C-Scans and the details of its steps are described below.

**Figure 11:**
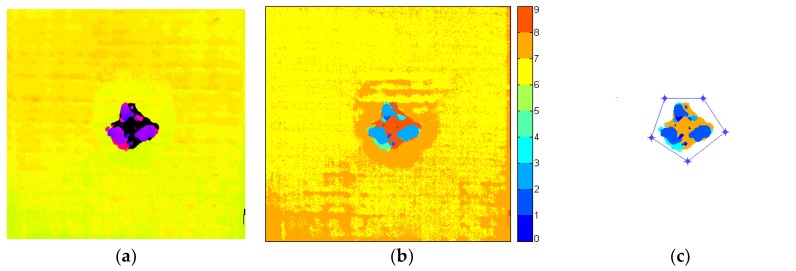
Selected steps of the C-Scan processing algorithm: (**a**) original C-Scan; (**b**) image after segmentation; (**c**) ROI selection after the background removal.

An original C-Scan image (see Figure 11a) consists of numerous values in the range of 0–255. The first algorithm stage is aimed at damage detection and consists of several steps. At the beginning, for the data reduction purpose, the C-Scan is segmented using a developed by the authors, dedicated to processing of ultrasonic scans, so-called Minima-Between-Peaks (MBP)-based image segmentation method, described in detail in [37]. This is a nonparametric histogram-based method, which relies on data reduction by selecting all detected local minima of a histogram of the processed image as the segmentation thresholds. In the resulting image (Figure 11b) with the significantly reduced number of classes (i.e., values, illustrated as different colors) it is easy to identify the values of layers representing the delaminated areas and the healthy regions of the specimen (background). The next step is to remove the background from the C-Scan by the selection and removal of the areas with the values that do not represent damage. Afterwards, morphological processing is applied in order to remove very small objects (connected components in a binary image) potentially being noise, which is performed using the so-called morphological area opening. Additionally, a region of interest (ROI) can be interactively selected by drawing a polygon around it (Figure 11c) and clearing the pixels being outside it in the case there are some other unwanted objects visible in the C-Scan.

**Figure 12:**
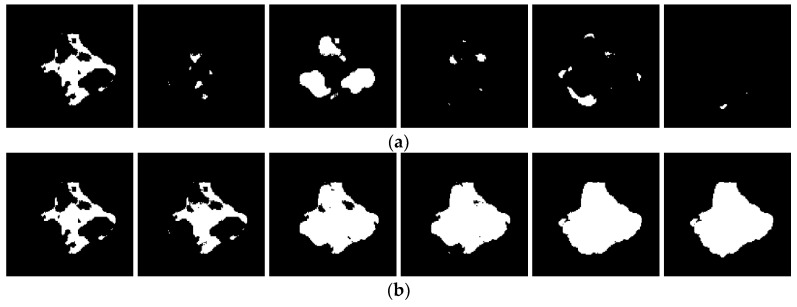
Detected damage layers at depths of, respectively: 0.51 mm, 0.78 mm, 0.94 mm, 1.25 mm, 1.37 mm, 1.92 mm: (**a**) based on the original result; (**b**) based on the filled result.

The second stage relates to 3D matrix building. For this purpose, calculations are made to assign the real values of depths of particular damaged composite layers. Firstly, the obtained values of classes (segments) in the segmented image are substituted by mode (most frequent) values that appear in the segments’ locations in the original C-Scan. This allows for the elimination of noise from the original C-Scan and obtaining planar images of all individual delamination layers. Then, their depth locations (in mm) are calculated based on known relation between the ToF C-Scan values and the real dimensions. Additionally, a mathematical relation is computed in order to estimate the depth location of the delamination identified in the dead zone (the black pixels in Figure 11a). Based on the knowledge that this region is near the top surface of the tested object, its depth is assumed to be calculated as the difference between the second closest to the top surface damage layer (i.e., the first with known depth location) and a mean difference between all the rest neighboring delamination layers. Figure 12a presents all the identified subsequent delamination layers in separate images. Optionally, similarly to the approach of the authors presented in [27], filling the pixels representing delamination with the increase of depth is implemented (see the result in Figure 12b). This is done by an iterative adding of successive damage layers of the 3D matrix. The assumption for such a filling follows from the geometric structure of BVID, i.e., the conically shaped damage in the thickness direction (see Figure 1), and the fact that C-Scans present incomplete information of damage due to covering delamination fragments by other delamination located above them. This approach, in turn, results in too much exaggeration of the damage reconstruction, but might be closer to the actual form of damage than in the case of the original result with the gaps originated from the C-Scan. Both results will be analyzed and compared with the actual form of damage reconstructed from the X-ray CT scans.

**Figure 13:**
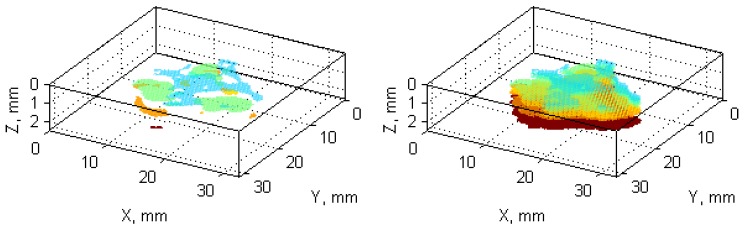

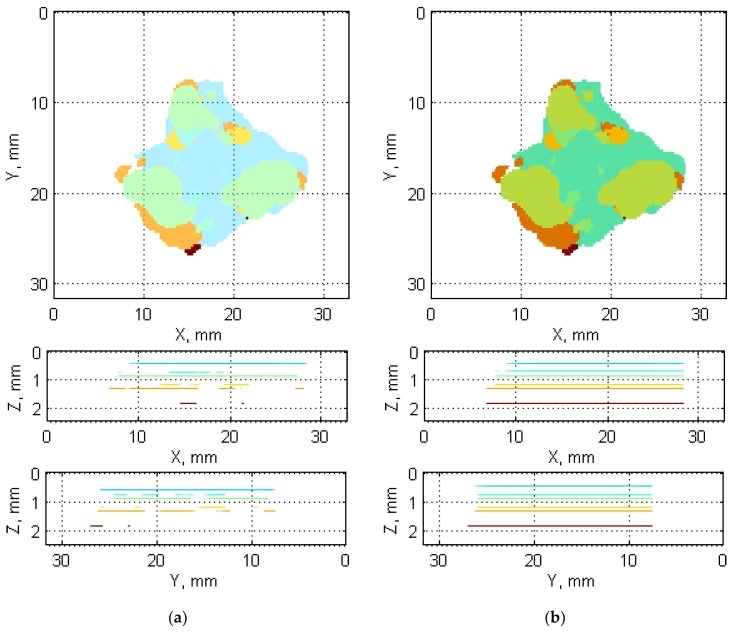
Visualization of the reconstructed damage based on UT C-Scans: (**a**) without gaps filling; (**b**) with gaps filling.

Finally, based on known scanning resolution, the pixel-mm ratio is calculated, and appropriate axes and labels are generated and assigned to the final 3D damage visualization (see Figure 13).

### 4.3. Reconstruction Algorithms for X-ray CT Scans

**Figure 14:**
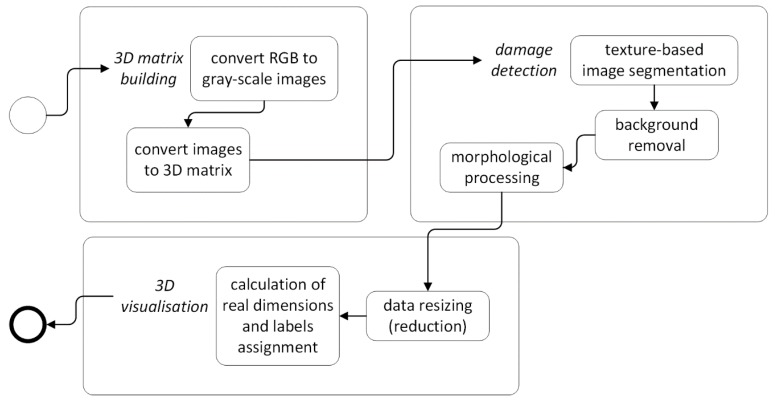
A general reconstruction algorithm for X-ray CT scans.

The developed damage reconstruction algorithm based on X-ray CT scans is summarized in a scheme shown in Figure 14.

**Figure 15:**
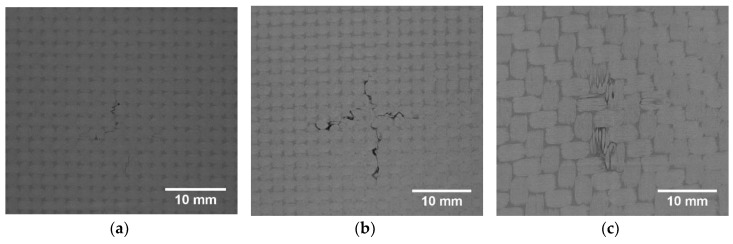
Representative X-ray CT scans with visible damage (from 250 slices): (**a**) no. 18; (**b**) no. 20; (**c**) no. 99.

Firstly, all raw X-ray CT sliced images (slices) are converted from RGB (see examples in Figure 15) to gray-scale images and then set together to form a 3D matrix. 

**Figure 16:**
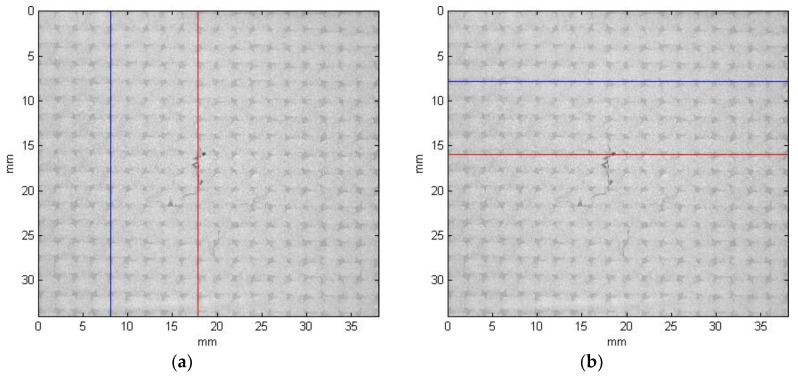
Positions of exemplary data vectors (not passing and passing through damage) in CT slices: (**a**) columns no. 368 and 814; (**b**) rows no. 357 and 726.

Then, each layer of the 3D matrix is processed separately in order to detect damage fragments through the whole composite thickness. When observed the raw CT slices one can notice a repetitive pattern (texture) within a particular image, which refers to the weave of the composite fibers. In order to detect damage fully automatically, a nonparametric texture-based image segmentation method was developed. The principle of operation of this algorithm was based on the assumption that any disorders of the image texture are considered to be damage. Due to the variable size of fibers visible in the CT slices over the composite thickness (cf. Figure 15a–c), the texture characteristics is determined for each slice separately. This is performed using several steps in a following way. Firstly, an authored adaptive thresholding with a variable threshold for each image column, and the same separately for each image row, is performed. A method of the selection of the thresholds is described below and the representative results, obtained for the slice presented in Figure 15a, are shown based on exemplary rows/columns marked in Figure 16.

**Figure 17:**
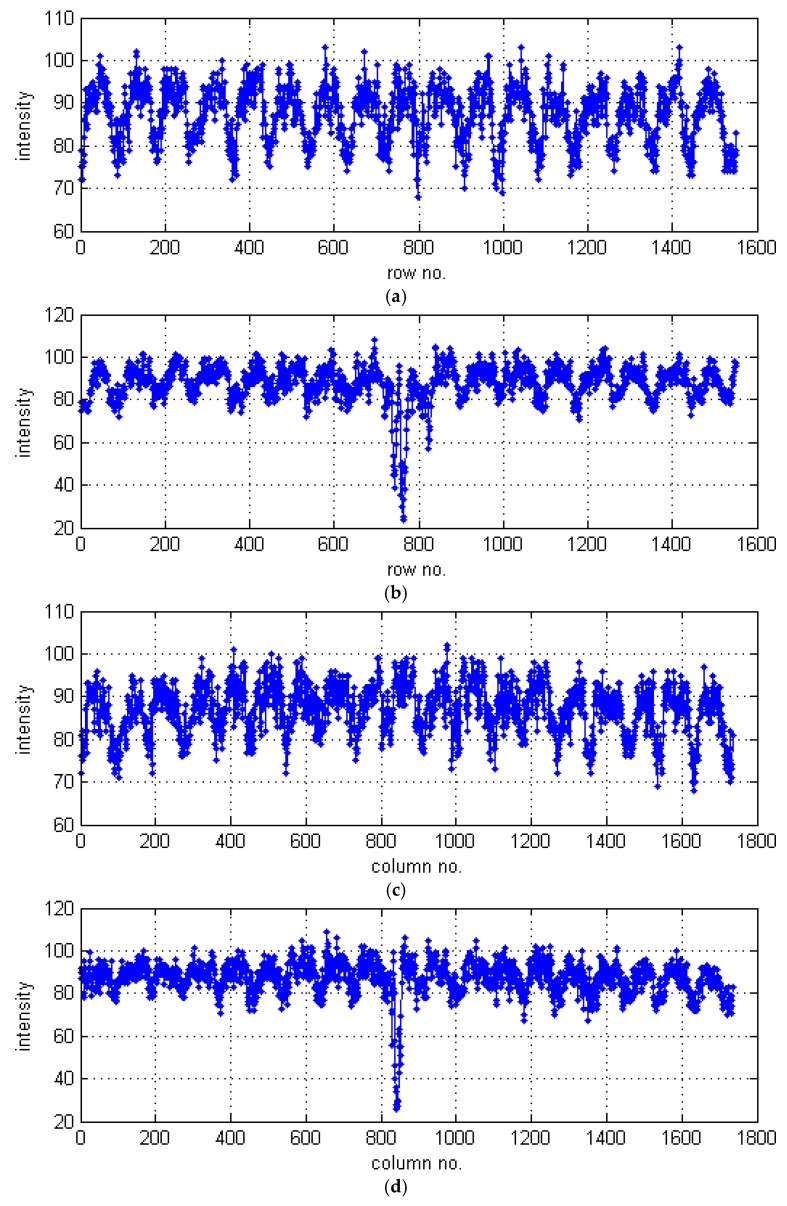
Data vectors extracted for selected CT image sections: (**a**) column no. 368; (**b**) column no. 814; (**c**) row no. 357; (**d**) row no. 726.

**Figure 18:**
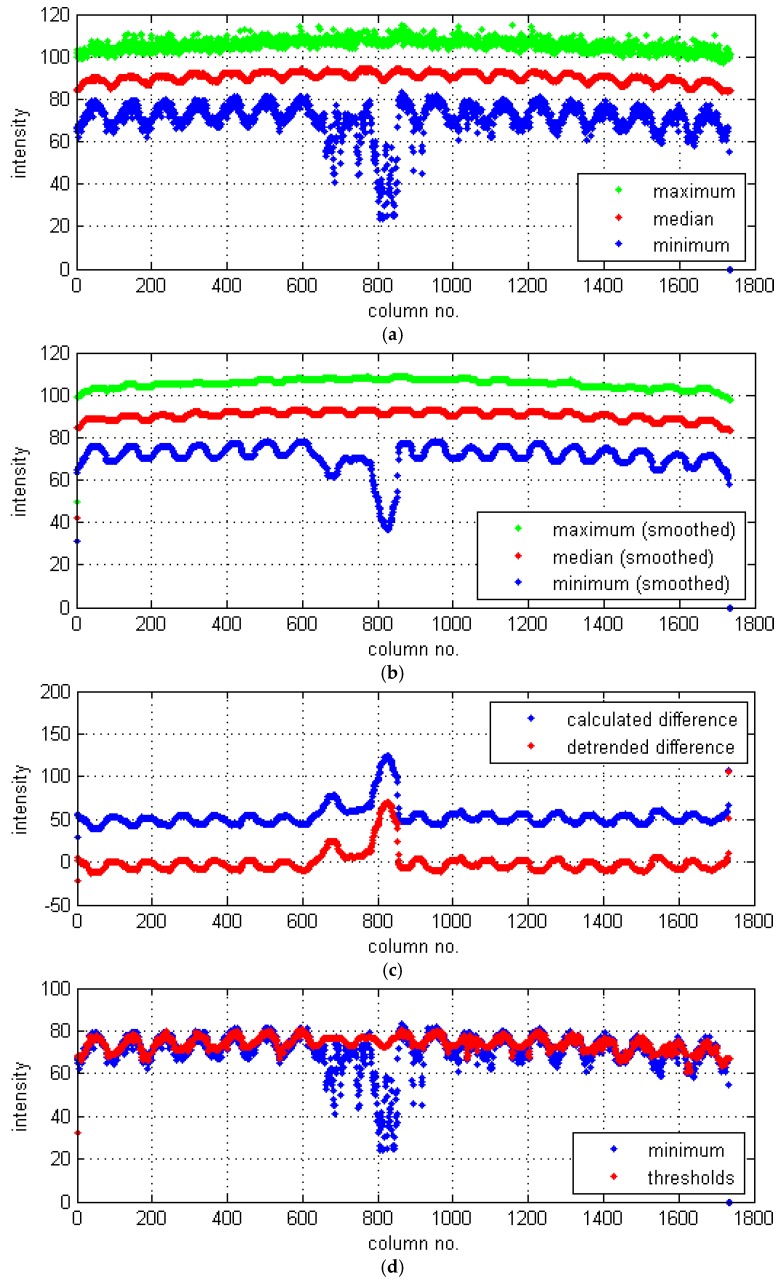
Column-based calculations of the texture characteristics of the CT slice: (**a**) statistical values; (**b**) statistical values after smoothing; (**c**) differences; (**d**) extracted threshold values.

For each image row/column, i.e., data vector with the intensity values (see the representative examples in Figure 17), their statistical values are calculated: minimum, median, and maximum (see the results set for all columns of the selected CT slice in Figure 18a). In Figure 17, it is well noticeable that pixels representing the areas affected by damage are in the range of lower intensities, and in Figure 18a, that the minimum values are sensitive to damage, whereas median and maximum values are unaffected by the texture anomalies.

The vector with minimum values is used as reference data to calculate the threshold values for the image binarization step. For this purpose, a model of the minimum vector (i.e., values of thresholds) is extracted from the original minimum values after corrections made using several steps. In order to detect all potential texture anomalies, including the intensities just slightly lower than the model texture, the minimum values vector is filtered using a one-dimensional median filter of a fourth order (selected experimentally), i.e., slightly smoothed. Then, in order to correct the vector fragments not matching to its overall trend, several calculations are made. The original vectors with the statistical values are significantly smoothed (see Figure 18b) by filtering them using a one-dimensional median filter of a 50th order (selected experimentally), which approximates the values to model ones. A sum of two differences based on the smoothed data is calculated between the median and minimum values as well as between the maximum and minimum values (see Figure 18c). Then, a detrending operation is applied to the result (Figure 18c), and the column positions with intensity values (after detrending) bigger than 0 indicate the positions that need replacing with another value. For these column cases, the threshold values are calculated as the median value minus a model (proper) difference between the median and minimum value (calculated as mean difference from these values for cases where the detrended difference is equal or lower than 0). The finally obtained threshold values for the image binarization step (different for each row/column, accordingly with the model texture) are presented in Figure 18d together with the original minimum values for comparison.

**Figure 19:**
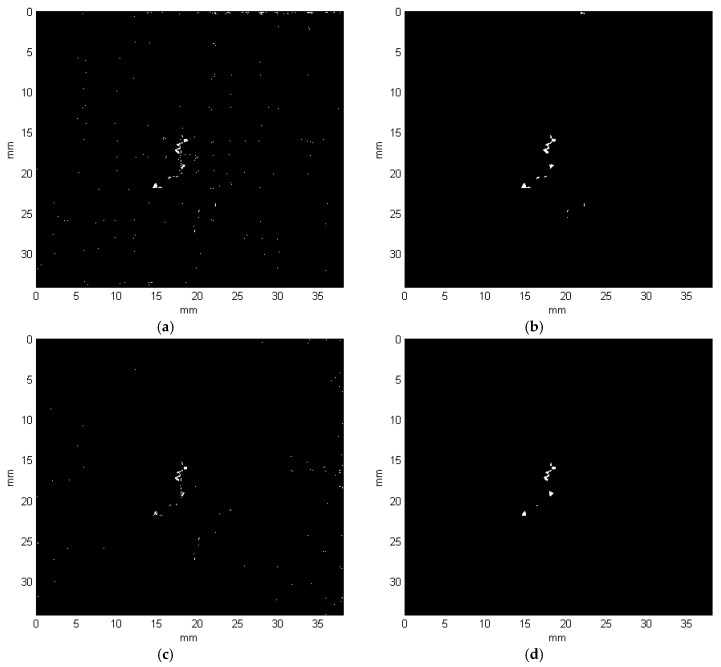
Binary results after thresholding using approaches: (**a**) column-based; (**b**) column-based after morphological processing; (**c**) row-based; (**d**) row-based after morphological processing.

The binarization using thresholds calculated based on image columns (see the result in Figure 19a) as well as based on rows (see Figure 19c) is performed for all CT slices individually, which allows for the automated background removal. Then, morphological processing is applied to the results in order to clean the remaining noise. This procedure consists of several steps selected experimentally, using known morphological operations, in sequence: bridging, setting majority, filling holes, clearing border, area opening, dilating, and eroding. The obtained results after morphological processing are presented in Figure 19b,d. 

**Figure 20:**
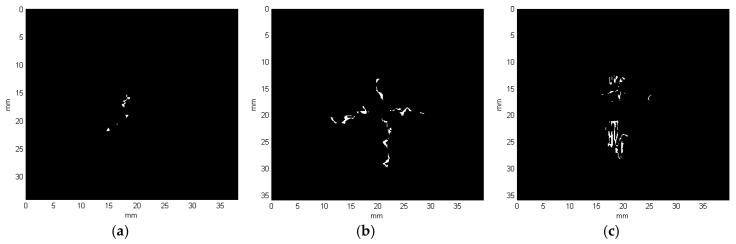
Binary results obtained for the representative X-ray CT slice cases: (**a**) no. 18; (**b**) no. 20; (**c**) no. 99.

Finally, common parts of these two results (i.e., the column-based and row-based) are extracted, which gives the final binary result of the detected damage layer. The final binary results for all the exemplary cases presented in Figure 15 are shown in Figure 20.

**Figure 21:**
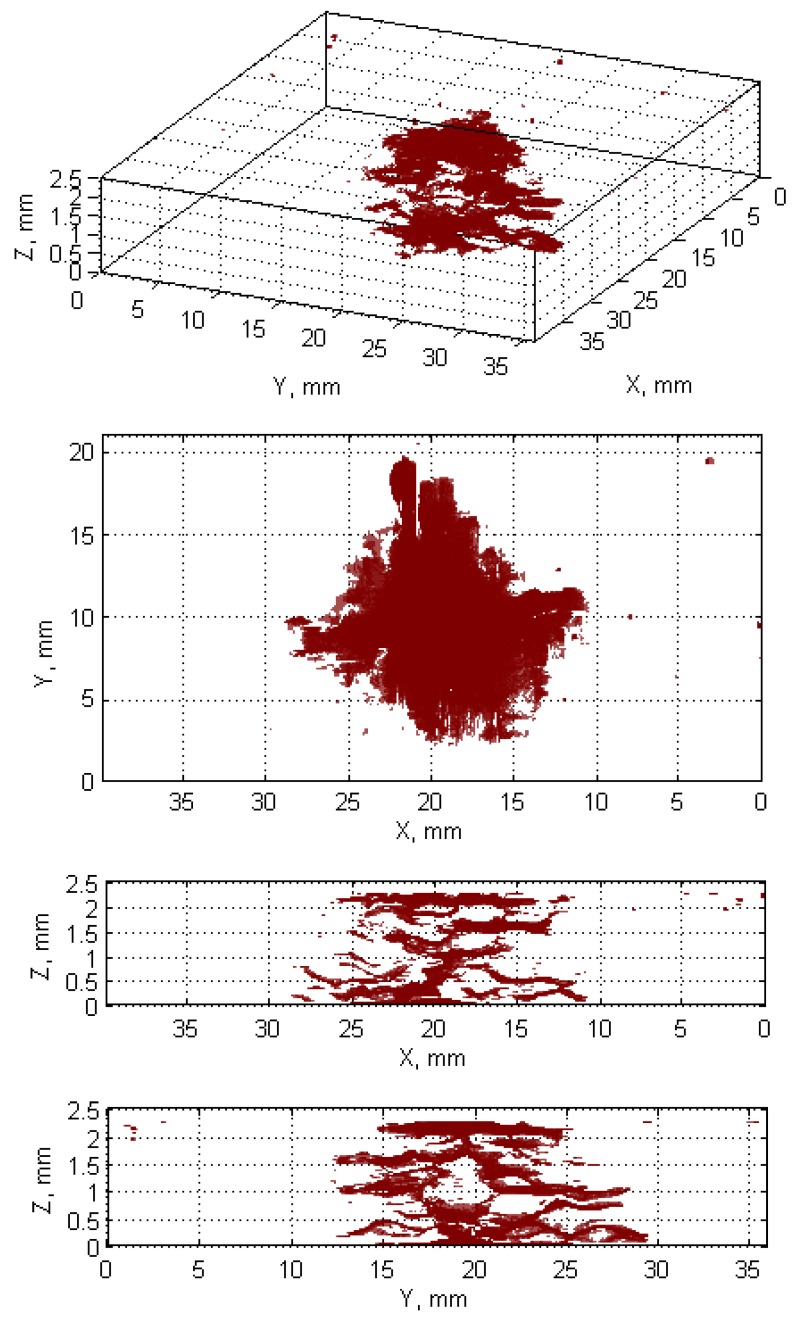
Visualization of the reconstructed damage based on X-ray CT scans.

Due to the very large data size, depending on used computing capabilities, it might be necessary to reduce the size of each resulting binary image before displaying the 3D damage visualization. In the last step of the algorithm, real dimensions of the resulting images are calculated based on known image resolution, step of slicing in the thickness direction, and resizing ratio (if the binary slices were resized), and the image axes with labels are appropriately adjusted. The 3D damage visualization is presented in Figure 21. Several first and last CT slices were excluded from this visualization due to a presence of too high level of scanning artefacts in the vicinity of the specimen external surfaces, which is typical for X-ray CT data [38]. 

## 5. Discussion and Conclusions

The obtained reconstructions show the damage distribution typical to BVID (see Figure 1), which is especially well visible on the obtained reconstruction from X-ray CT scans (cf. Figure 21). The small discrepancies between a model of BVID and the registered true BVID may result from the influence of the weave onto damage propagation mechanisms, thickness of the tested specimen, and inaccuracies and errors of NDT and further processing. The obtained UT reconstructions (presented in Figure 9 and Figure 13) reveal more significant differences with respect to the model of BVID, which is expected due to their much lower resolution with respect to the X-ray CT. The reasons of these differences are the same as for X-ray CT reconstructions. Nevertheless, it reveals the characteristic pine tree shape of the BVID.

Since all the damage reconstructions were performed using different types of data originated from the results of NDT methods exploiting different physical phenomena, it is difficult to compare them directly. However, the authors performed a qualitative, i.e., visual, as well as an attempt of a quantitative analysis of the obtained results.

When analyzed the reconstruction results visually, it was noticed that the results of UT, both in B-Scan and C-Scan modes, are a reliable source of the diagnostic information about BVID presence in the tested object since the multi-level character of such a type of damage is well detectable. The 3D damage visualization gives much enriched information than the raw UT results themselves, since the damage distribution over the composite thickness can be easily observed and analyzed. An online display of such a visualization during inspections could be useful for UT operators and helpful in fast identification of the existence of BVID or another type of damage.

When comparing the reconstruction results obtained using the phased-array (B-Scans) and pulse-echo (C-Scans) UT techniques, it was noticed that the latter allowed obtaining more details about the shape of damage, including a number of damage layers and their extent (cf. Figure 9 and Figure 13). However, this fact results from the problems connected with the influence of the applied UT technique and its parameters on the obtained results as well as their dependences with the defect characteristics and geometrical properties of the tested object. In this experiment, the results of the phased-array UT method are less satisfactory due to the problem of the small thickness of the tested composite specimen, which, in the case of the pulse-echo method, was partially avoided owing to the used transducer with the delay line. This problem with detection of near-surface defects did not appear in the previous authors’ studies [28], where a thicker specimen (4 mm) was examined. As mentioned, the reconstruction based on C-Scans from the pulse-echo method brought more complete results in this experiment and its great similarity with the reference data reconstructed from the X-ray CT data can be noticed (cf. Figure 13 and Figure 21). Nevertheless, both the reconstruction results allowed for the identification of damage at several specimen’s depths, which can be referred to delamination and some matrix cracking areas. However, the skew and transverse cracks are not distinguishable in the UT data and the detected damage should be mainly assigned to delamination. The round indentation on the specimen’s surface resulting from the shape of the impactor tip cannot be visible in the ultrasonic data, which results from the limitation of the UT method to detection of only internal defects. Marks similar to the cross-shaped crack near the top surface of the specimen (visible by the naked eye, see Figure 3, and in CT scans, see Figure 15a,b) can be identified in the reconstruction made based on C-Scans (first layer from the top, visible in Figure 12 and Figure 13), whereas one can notice their absence above the first damage layer reconstructed based on B-Scans (see Figure 9) due to the reason of the problem with the near-surface defects detection, explained before.

Another conclusion about the real shape of the introduced BVID can be made when observed the reference CT reconstruction results, namely the damage layers (see the side views in Figure 21) are similar to the scheme presented in Figure 1, i.e., multiple delamination with matrix cracks are detectable that spread along with the depth of the specimen, however, there remain some unaffected areas inside the pine tree distribution damage. For that reason, one can also conclude that the C-Scan-based damage reconstruction with the version without gaps filling (Figure 12a and Figure 13a) results in obtaining an incomplete image of damage, whereas the version with gaps filling (Figure 12b and Figure 13b) seems to give this image overestimated with respect to the real damage. The performed quantitative analysis, summarized below, puts more information to this comparison. Moreover, owing to the highest accuracy of the X-ray CT method, one can additionally observe a waviness of the delaminated areas resulting from the weave of the composite fibers, which is obviously impossible to observe in the results of other less accurate NDT methods, where they are flatter.

**Figure 22:**
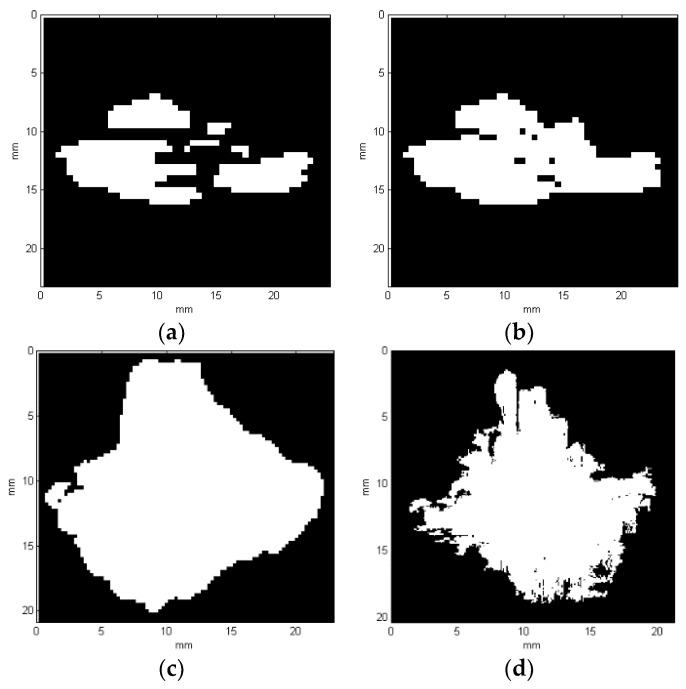
Planar projections of the reconstructed damage based on: (**a**) B-Scan (the first version); (**b**) B-Scan (the second version); (**c**) C-Scan; (**d**) X-ray CT scan.

Based on known resulting image resolutions and calculated thicknesses of the layers, the volume of the detected damage regions in mm^3^ as well as the area of planar projection (see Figure 22) in mm^2^ with other geometrical properties were calculated. The results obtained based on different NDT data are summarized in Table 1.

The analysis of the quantitative data stored in Table 1 indicated that the real volume of damage (assumed based on the X-ray CT data) is the closest to the damage reconstructed based on a C-Scan without gaps filling, whereas other reconstructions reveal a significant oversizing. Thus, additional measures were established to compare geometrical properties of the planar (top) damage view: surface area, equivalent diameter, and major axis length. The calculation of the area of planar projection based on C-Scan reveals oversizing, which often takes place during UT data analysis due to a diameter of the ultrasound beam [9], whereas it is undersized in the case of the B-Scan-based reconstruction. The equivalent diameters and major axis lengths are in all the cases comparable, but the first measure is oversized in the C-Scan and undersized in the B-Scan, whereas the second measure is oversized for all the reconstruction types, however, these differences are relatively small.

The presented results show that it is difficult to select the one appropriate NDT data type and reconstruction method that could dimensionally map the damage. However, summing up the results of qualitative and quantitative analyses, it can be concluded that the C-Scan reconstruction without gaps filling brought the best results. The authors noticed that such the reconstruction with only a partial gaps filling could be a better solution, which is planned to be developed in further studies.

The results presented in this paper are a part of on-going research studies, and it is planned to perform a quantitative analysis, including results from FE simulations, based on the developed algorithms and a larger representation of experimental data. However, it should be underlined that the results obtained within this study allowed to gain knowledge on sensitivity of particular NDT methods to BVID and create further possibilities of simulation of BVID in order to predict structural residual life basing on computational models only.

## Figures and Tables

**Table 1:** Quantitative data of the reconstructed damage based on different NDT results.

-	X-ray CT Scan	C-Scan		B-Scan	
		Unfilled	Filled	First Version	Second Version
volume (mm^3^)	13.2	9.5	44.5	23.4	58.8
area of planar projection (mm^2^)	151.6	242.5		87.0	117.7
equivalent diameter (mm)	13.9	17.6		10.5	12.2
major axis length (mm)	15.9	19.2		22.5	21.0
